# Rastermap: a discovery method for neural population recordings

**DOI:** 10.1038/s41593-024-01783-4

**Published:** 2024-10-16

**Authors:** Carsen Stringer, Lin Zhong, Atika Syeda, Fengtong Du, Maria Kesa, Marius Pachitariu

**Affiliations:** Howard Hughes Medical Institute Janelia Research Campus, Ashburn, VA USA

**Keywords:** Computational neuroscience, Neural encoding

## Abstract

Neurophysiology has long progressed through exploratory experiments and chance discoveries. Anecdotes abound of researchers listening to spikes in real time and noticing patterns of activity related to ongoing stimuli or behaviors. With the advent of large-scale recordings, such close observation of data has become difficult. To find patterns in large-scale neural data, we developed ‘Rastermap’, a visualization method that displays neurons as a raster plot after sorting them along a one-dimensional axis based on their activity patterns. We benchmarked Rastermap on realistic simulations and then used it to explore recordings of tens of thousands of neurons from mouse cortex during spontaneous, stimulus-evoked and task-evoked epochs. We also applied Rastermap to whole-brain zebrafish recordings; to wide-field imaging data; to electrophysiological recordings in rat hippocampus, monkey frontal cortex and various cortical and subcortical regions in mice; and to artificial neural networks. Finally, we illustrate high-dimensional scenarios where Rastermap and similar algorithms cannot be used effectively.

## Abstract (web summary)

Rastermap is an analysis method for exploring dynamical and spatial relationships among hundreds to hundreds of thousands of neurons. The algorithm uses a fast optimization technique to discover complex neural patterns, such as sequences.

## Main

High-density electrodes and two-photon calcium imaging have generated an explosion of large-scale neural recordings^1,2^. Visualizing and analyzing such recordings can be done either directly at the single-cell level^3,4^ or at the population level using dimensionality reduction methods^5–8^, but both methods have caveats. Visualizing neurons one at a time can be difficult because single neurons are often very noisy^9,10^. Furthermore, single-neuron visualizations cannot show the population-wide coordination of neural firing patterns, which can vary across trials, leading to ‘trial-to-trial’ variability^11–13^. On the other hand, dimensionality reduction algorithms can find common patterns of covariation across neurons, allowing further analyses to be restricted to just these reliable modes of activity. However, in large-scale recordings or in recordings with complex tasks, many components must be used to capture the high-dimensional structure of the neural activity patterns^14–18^.

Nonlinear dimensionality reduction methods can overcome some of these limitations. For example, manifold discovery algorithms such as t-distributed stochastic neighbor embedding (t-SNE) and uniform manifold approximation and projection (UMAP) embed the firing patterns of neurons into one or two dimensions^19–21^. Such algorithms can be used, for example, to place neurons with similar firing patterns close to each other. However, these algorithms are typically used to visualize the embedding space, which is a visualization of the relations between neurons rather than a direct visualization of their activity patterns^22^. Furthermore, it can be challenging for these algorithms to maintain both local and global structure on neural data, as their cost functions are not optimized for such data. Methods such as t-SNE and UMAP can also suffer from local minima during optimization^23^, and it can be difficult to evaluate what constitutes true clustering in the embedding space and what is an artifact of the algorithms^24^.

**Extended Data Fig. 1:**
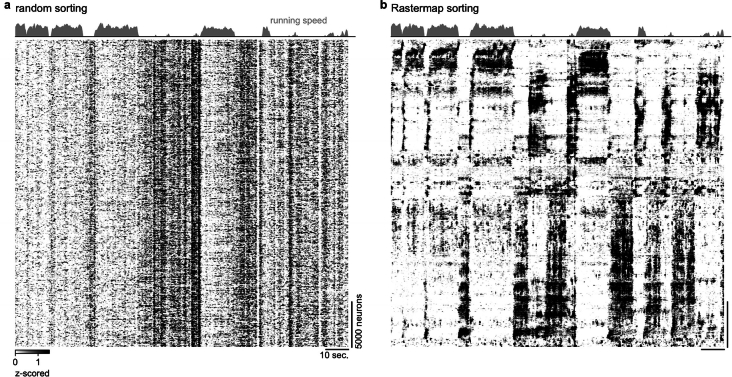
Spontaneous neural activity in random order and sorted by Rastermap. **a**, Same neural activity as Fig. 3, with random sorting. **b**, Fig. 3b reproduced for comparison.

**Extended Data Fig. 2:**
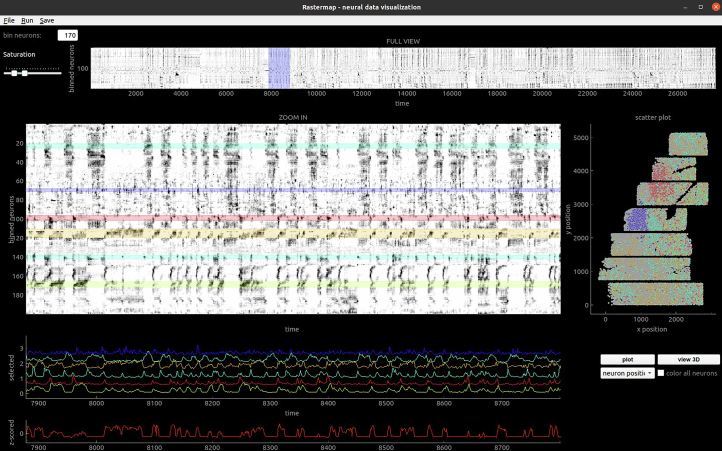
Rastermap graphical user interface (GUI). 1st row: Full recording, with selected time period shown in blue. 2nd row left: Superneuron activity by time in selected time period, with user-selected clusters highlighted in different colors. 2nd row right: All neurons shown in gray, and neurons from selected clusters shown in color. 3rd row: Mean activity in each of the user-selected clusters. 4th row: Behavioral/task variable plotting area showing the mouse’s running speed.

Unlike these existing methods, Rastermap provides a structured visualization of the activity patterns across different groups of neurons, illustrating how these activity patterns relate to each other. The Rastermap visualization is inspired by ‘classical’ population raster plots, where the spike train of each neuron is shown as a row of rasterized ticks, often alongside other variables such as behavior^25^. These raster plots can illustrate the average population activity; to improve the plots, one can reorder the neurons across the *y* axis of the plot so that nearby neurons have similar activity patterns (Extended Data Fig. 1). Our reordering algorithm, Rastermap, is optimized for neural data by combining two commonly observed features of neural activity: (1) a power law scaling of eigenvalue variances and (2) sequential firing of neurons. We demonstrate here that Rastermap outperforms t-SNE, UMAP and other nonlinear dimensionality reduction methods on simulations of neural data. The algorithm is also fast: it runs in less than 2 min on datasets with tens of thousands of neurons. Rastermap is implemented in Python and can be run in a Jupyter notebook, on the command line or in the provided graphical user interface (Extended Data Fig. 2).

## Results

**Fig. 1:**
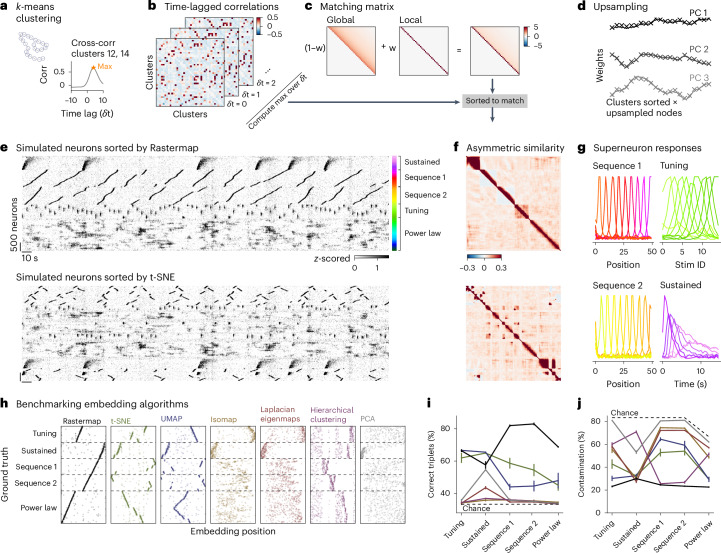
Benchmarking Rastermap on simulated data with multiplexed neural activity. **a**–**d**, These panels illustrate how Rastermap works. **a**, First, Rastermap divides neurons into 50–200 clusters based on their activity (left). The cross-correlations between different clusters are computed at several time lags (right). **b**, The cluster correlations at different positive time lags are shown for a subset of clusters, and the entry-wise maximum of these matrices over a time window from 0 to *T*_max_ defines an ‘asymmetric similarity matrix’. **c**, The asymmetric similarity matrix is sorted to match the ‘matching matrix’, which is a sum of a global similarity matrix and a local similarity matrix. **d**, The cluster features are upsampled using a locally linear interpolation method, and then each neuron is assigned to an upsampled cluster center. **e**, The simulated neurons were sorted by Rastermap or t-SNE and then averaged in bins of 30 neurons—the averages of these neurons are called ‘superneurons’. **f**, The sorted asymmetric similarity matrix for the simulation. **g**, The activity of the superneurons aligned to different stimulus events. **h**, The sorting of neurons from various algorithms plotted against the ground truth sorting. **i**, For each module of the simulation and each algorithm in **h**, the percentage of correctly ordered triplets is shown (*n* = 10 simulations; error bars represent s.e.m.). **j**, The percentage of contamination in a module with neurons from other modules (*n* = 10 simulations; error bars represent s.e.m.). Corr, correlation; stim, stimulus.

The goal of Rastermap is to obtain a sorting of all neurons in a recording, such that nearby neurons in the sorted list have similar functional properties, and, overall, the neural pairwise similarity decays smoothly as a function of pairwise distance in the sorting. Equipped with this sorting, we can make a single raster plot of all neurons that visualizes the most common patterns of activity. We typically use the full recording session to compute the Rastermap sorting, but we also show examples of Rastermap on trial-based data below. To start, we cluster the neural activity profiles by *k*-means clustering, typically into *N*_clusters_ = 100 distinct clusters (Fig. 1a). We then define an asymmetric similarity measure between clusters, as the peak cross-correlation between the cluster activities at non-negative time lags (Fig. 1a,b). The asymmetry induced by this metric ensures that a well-defined ordering can be achieved, so that clusters with earlier activity are typically displayed toward the bottom of the raster plots.

**Extended Data Fig. 3:**
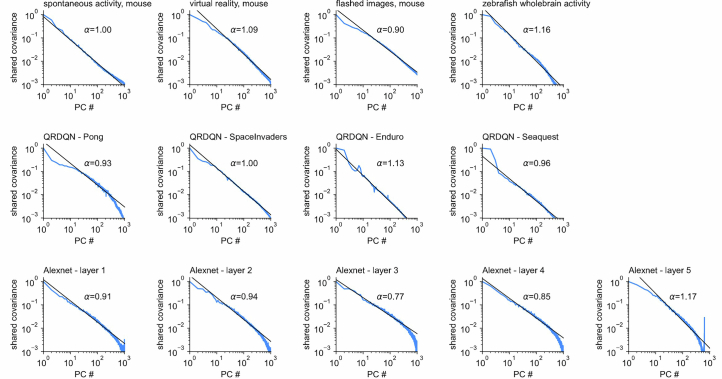
Power-law of shared variance of neural activity. We ran SVCA (shared variance components analysis, ref. ^16^) on the large-scale neural recordings and artificial neural network activity analyzed in this study (blue line), and fit the best 1/n^*α*^ line to each spectrum using points from 10 to 500 (black). We note this estimation procedure uses all timepoints, and is not a measure of stimulus-driven dimensionality scaling like the cvPCA method from ref. ^17^.

Having obtained an *N*_clusters_ by *N*_clusters_ similarity matrix, the optimization goal of Rastermap is to permute the rows and columns of this matrix until it matches a predefined matrix as closely as possible. The predefined matrix is chosen as a sum between a global and a local similarity matrix (Fig. 1c; see also multi-perplexity t-SNE^26,27^). The global similarity matrix has a heavy-tailed distribution that decays smoothly as a function of distance between clusters, with an eigenvalue decay of 1/*n*, which is observed in neural recordings and artificial neural networks (Extended Data Fig. 3). The local part of the matrix has a ‘traveling salesman’ structure^28^, where the similarity is only high between consecutive nodes in the sorting to capture sequential activity patterns observed in neural datasets. The local and global matrices are added together with a weighting term *w*, the locality parameter, that can be adjusted based on the properties of the data.

The resulting matching matrix is the target that must be matched to the neural similarity matrix by permuting rows and columns, but the user can also input their own matching matrix, for example if a smoother representation is desired. At every iteration of the optimization, we exhaustively check if any consecutive sequence of *N* clusters can be moved to any other position in the sorting, starting with sequences of length 1 and then progressively checking longer sequences, extending beyond 2-length and 3-length sequences, which are often used^29,30^. This specialized optimization can be implemented efficiently on modern CPUs using the numba Python package^31^, as long as *N*_clusters_ ≤ 200.

After re-sorting, the neural similarity matrix resembles the matching matrix, as illustrated on an example simulation in Fig. 1f. Having obtained an ordering for the clusters, we must now obtain an ordering for the neurons. To do this, we upsampled the sorted cluster activities by a factor of 10 in the principal component analysis (PCA) feature space, thus creating *N*_clusters_ × 10 positions that can be matched to single neurons (Fig. 1d). Single neurons were then assigned to the position that is most highly correlated to their activity in PCA space. When the number of neurons is very large (thousands or more), we cannot visualize them as rows of a Rastermap due to a lack of vertical pixels on most monitors. We, therefore, bin the thousands of neurons into hundreds of ‘superneurons’. Superneurons are averages across groups of neurons that were put next to each other in the Rastermap, which, by definition, have similar firing patterns. An added bonus of creating superneurons is that they have less noisy activity compared to single neurons^32^.

### Benchmarking with known ground truth

Benchmarking visualization methods is difficult because a good visualization should be evaluated based on its ability to simplify complex data, and this is difficult to measure for real datasets. The approach that we take here is to start with a realistic simulation of neural activity, which contains multiple, complex signals with different spatiotemporal signatures. We then randomly shuffle the neurons and ask different methods to undo the shuffling. The simulated populations contain multiple sub-modules with realistic firing patterns: we use two modules with sequential firing, modeling, for example, place cells when an animal runs through a linear corridor; we then add a module of sensory responses to repeated flashed stimuli where the neurons have wide tuning curves to these stimuli; we also add a module of neurons with different response durations and latencies to a single stimulus presented many times; finally we add a module of neurons with power law PCA structure and add small amounts of this module to all other modules, to model the effect of spontaneous, ongoing activity as correlated noise across the population^16^ (Fig. 1e). Note that, if an algorithm sorts neurons from a module according to their power law contribution, this would be considered incorrect in our benchmark, unless those neurons are in the power law module.

**Extended Data Fig. 4:**
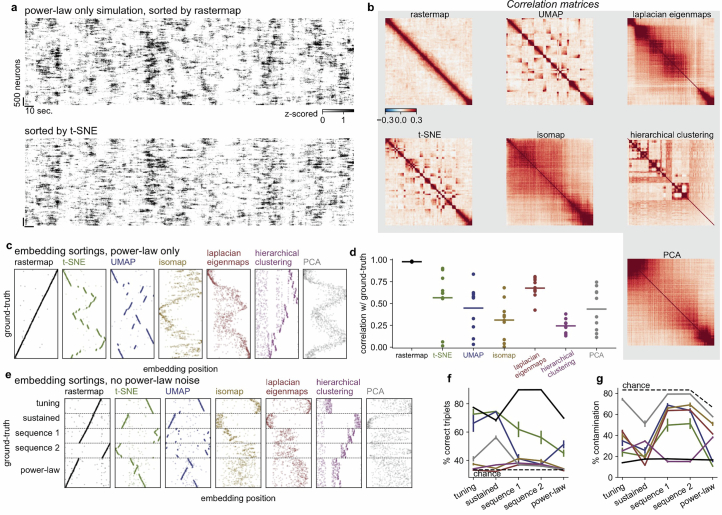
Power-law simulation only (global structure). We simulated a power-law module with 6,000 neurons. **a**, The simulated neurons were sorted by Rastermap or t-SNE, then averaged in bins of 30 neurons, which we call superneurons. **b**, The correlation matrices for the superneurons computed from various algorithms. **c**, The sorting of neurons from various algorithms plotted against the ground-truth sorting. **d**, The absolute correlation of the algorithm sorting with the ground-truth sorting (n=10 simulations, line represents mean). **e**-**g**, Same as Fig. 1h–j, but we did not add noise from the power-law module to neurons in the other modules in the simulations (error bars represent s.e.m., n=10 simulations).

Rastermap was able to find the natural ordering of this simulation, whereas other methods, such as t-SNE, failed, typically oversplitting clusters and positioning the pieces far from each other (Fig. 1e). After sorting, the asymmetric similarity matrix contained high values closer to the diagonal in Rastermap compared to other methods, such as t-SNE (Fig. 1f). The superneurons, defined as averages of 50 consecutive neurons in the Rastermap sorting, have clearly defined tuning properties, whether as part of a sequence or in response to the simulated stimuli (Fig. 1g). We also simulated neurons from a power law module only and found that Rastermap produced a more smoothly decaying correlation matrix compared to other methods (Extended Data Fig. 4a,b).

**Extended Data Fig. 5:**
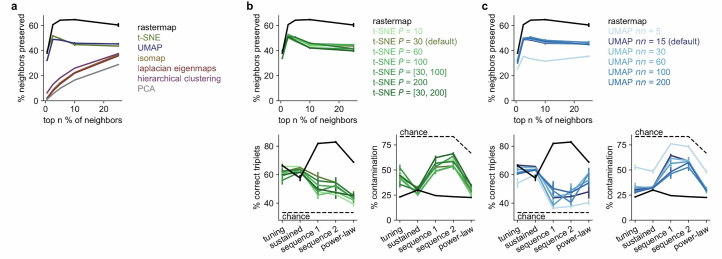
Benchmarking embedding algorithms. **a**, The KNN score for benchmarking embedding algorithms from ref. ^26^: the percentage of *k*-nearest neighbors in the original space that are preserved as *k*-nearest neighbors in the embedding space (error bars represent s.e.m., n=10 simulations, for all panels). **b**, Top: Same as a for t-SNE embeddings computed with various perplexities, including multiple perplexities^27^. Bottom: Like in Fig. 1i,j, percentage of correct triplets and percentage contamination for the different t-SNE embeddings. **c**, Same as **b** for UMAP embeddings computed with various values of n_neighbors (called nn in the legend).

To benchmark a set of commonly used embedding algorithms, we compare their embedding order with that of the ground truth (Fig. 1h). Although there are no relations between modules by construction, within each module we expect a non-interrupted monotonic relation between the embedding position and ground truth. To quantify the similarity of these orderings, we use two measures: the number of correctly ordered triplets and the percent contamination of neuron groups from the same module. The fraction of correctly ordered triplets was higher for Rastermap across the two sequence modules and the power law module compared to all other methods. Rastermap, t-SNE and UMAP performed similarly on the flashed stimulus response modules (tuning and sustained), we suspect due to the wider tuning of the single neurons in these modules resulting in lower dimensionality (Fig. 1i). Finding correctly ordered triplets is not sufficient to ensure a good ordering; these triplets also have to be part of a continuous, unbroken module. To estimate how broken up a module is, we quantified the percent contamination with other modules for the neurons sorted in between any two neurons from the same module (Fig. 1j). This contamination was lowest for Rastermap across all modules except the sustained module in which all algorithms performed similarly. Additionally, we showed that Rastermap also performs better on the *k*-nearest neighbor metric introduced by ref. ^26^ (Extended Data Fig. 5). We also benchmarked Rastermap on simulations with a power law module only and without power law noise added to each neuron, and we found that Rastermap also outperformed other methods in these cases (Extended Data Fig. 4c–g).

**Extended Data Fig. 6:**
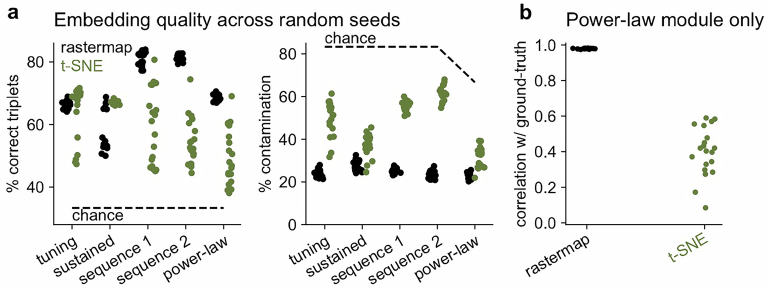
Reproducibility of sorting. **a**, We ran Rastermap and t-SNE with 20 different random seeds to sort the simulation from Fig. 1e–h with five modules. Percent correct triplets and percent contamination are reported. **b**, 20 sortings of the power-law module only simulation from Rastermap and t-SNE with different random seeds.

**Extended Data Fig. 7:**
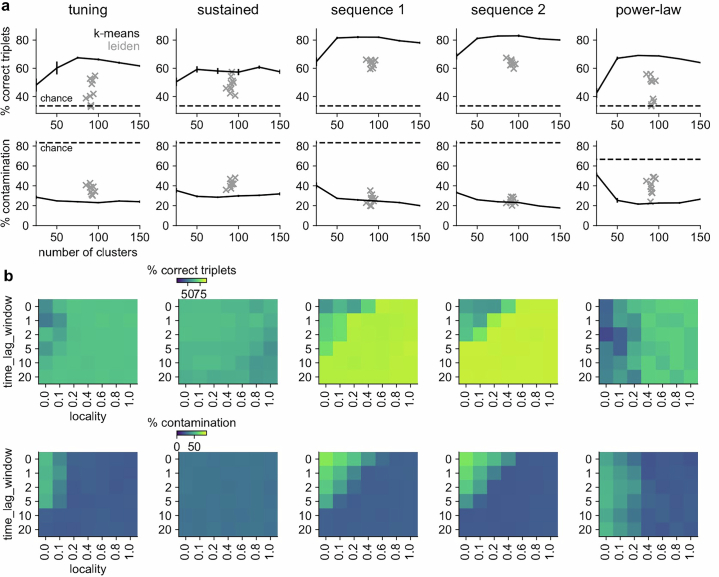
Rastermap embedding quality as a function of parameters. In Fig. 1, we ran Rastermap with 100 clusters (k-means clustering), locality parameter of 0.8, and time_lag_window of 10. Here we run Rastermap with varied parameter values. **a**, Rastermap was run with different numbers of clusters (black) for each of the ten simulations from Fig. 1 (error bars represent s.e.m.). We also ran Rastermap using the Leiden algorithm to perform clustering with a resolution of 3.0 and 100 neighbors, which produced 100 clusters (gray) – this clustering method performed worse than k-means. Percent correct triplets, percent contamination and chance level (dashed line) computed as in Fig. 1i,j. **b**, Average percent correct triplets and percent contamination computed from Rastermap sorting from the ten simulations, using different locality and time_lag_window values.

Finally, we evaluated the consistency of Rastermap and t-SNE across multiple runs with different random seeds. The embedding quality across runs varied less for Rastermap than it did for t-SNE (Extended Data Fig. 6). We noticed that the main source of variability in Rastermap is from the initial clustering procedure, and we evaluated whether other more stable clustering algorithms perform better. However, graph-based clustering methods, such as the Leiden algorithm, performed substantially worse (Leiden^33^; Extended Data Fig. 7). Nonetheless, it is possible for users to potentially input other clustering algorithms to Rastermap^34^. We also found that the embedding quality was robust across various Rastermap parameters, suggesting that the user does not need to be precise when testing parameters for the visualization (Extended Data Fig. 7).

### Rastermap on 50,000 neuron recordings during virtual reality

**Fig. 2:**
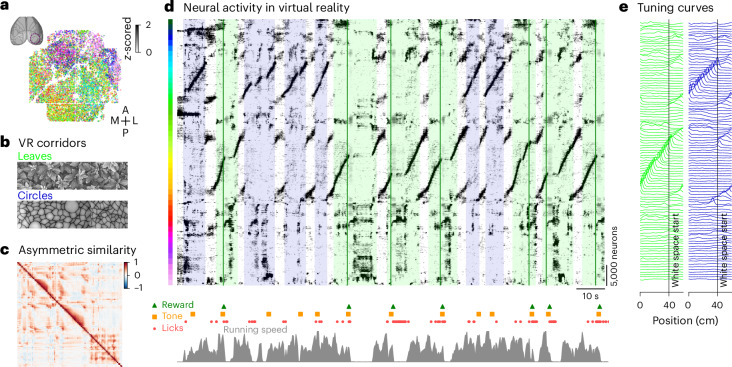
Applying Rastermap to neural activity from a virtual reality task. **a**, In total, 66,318 neurons were recorded across mouse visual cortex using two-photon calcium imaging, colored by position in the Rastermap sorting. **b**, During the recording, mice navigated through a one-dimensional virtual reality (VR) with two different corridors (‘leaves’ and ‘circles’) that were separated by a gray area and randomly interleaved. A tone was played in each corridor at a random time, and, in the ‘leaves’ corridor, the tone was followed by a reward. **c**, The sorted asymmetric similarity matrix from the recording. **d**, Top: neural activity sorted by Rastermap; colored backgrounds denote the type of corridor; green lines denote rewards. Bottom: event times in the task and running speed. **e**, Superneuron tuning curves to positions along each corridor. M, medial; A, anterior; P, posterior; L, lateral.

To illustrate Rastermap in practice, we apply it to a variety of datasets. We start in this section and the next with datasets collected in our own laboratory, using two-photon calcium imaging of large neural populations of up to 70,000 simultaneously recorded neurons at sampling frequencies of approximately 3.2 Hz^35,36^. First, we applied Rastermap to data collected in visual cortex during navigation and sensory decision-making in virtual reality (Fig. 2a)^37–39^. Mice were trained to run through two corridors with different naturalistic textures on the walls (‘leaves’ and ‘circles’) (Fig. 2b). Reward was delivered at pseudo-random positions in the leaves corridor, after an auditory cue, and the mouse had to lick to trigger the reward. After a few weeks of training, the mouse learned to reliably lick only in response to the cue in the leaves corridor and not in response to the cue in the circles corridor.

**Extended Data Fig. 8:**
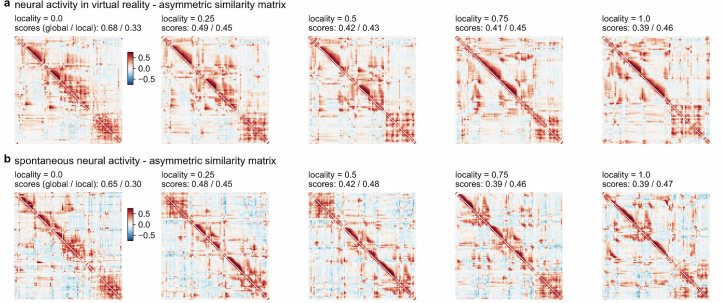
Rastermap sorting as a function of the locality parameter. **a**, Asymmetric similarity matrix from neural activity from a virtual reality task, for different values of the locality parameter (0.75 used in Fig. 2d). **b**, Same as a for spontaneous activity (locality = 0 used in Fig. 3b).

**Extended Data Fig. 9:**
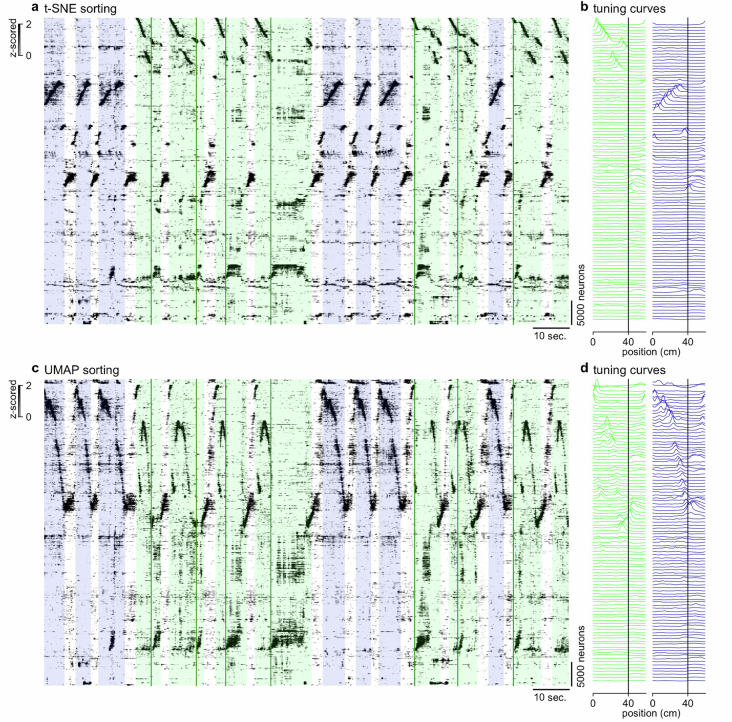
Neural activity from a virtual reality task sorted by t-SNE and UMAP. **a**-**b**, Same as Fig. 2d–e using sorting from the t-SNE algorithm. **c**-**d**, Same as Fig. 2d–e using sorting from the UMAP algorithm.

The neural activity generated in this task followed clear sequential patterns, which Rastermap was able to group together (Fig. 2c,d). Two large populations of neurons can be seen encoding the circles and leaves corridors, with a slightly larger population encoding the rewarding corridor (Fig. 2e). To place the sequences for the corridors together in the sorting, a non-zero locality weight was required (Extended Data Fig. 8a)—t-SNE and UMAP did not succeed at this (Extended Data Fig. 9). We also observed populations that encode the gray space, an area between corridors without visual stimuli. The encoding of the gray space was also sequential as a function of position and mostly did not depend on either the previous or the next corridor. The sequential activity was interrupted in the leaves corridor at times when the mouse stops to collect the reward.

There are also multiple reward-related populations of interest visible in just the single plot from Fig. 2. In a separate study, we found that one of these populations at the top of the Rastermap was active in the rewarded corridor only before the reward was delivered, turning off after reward delivery, suggesting that those neurons encode reward probability^40^. We discovered this population of neurons using Rastermap, illustrating that hypothesis generation is possible with this visualization technique. Finally, there are other populations of neurons that do not seem engaged by any aspects of the task, which we think is related to the spontaneous orofacial behaviors.

### Rastermap on neural recordings during spontaneous behaviors

**Fig. 3:**
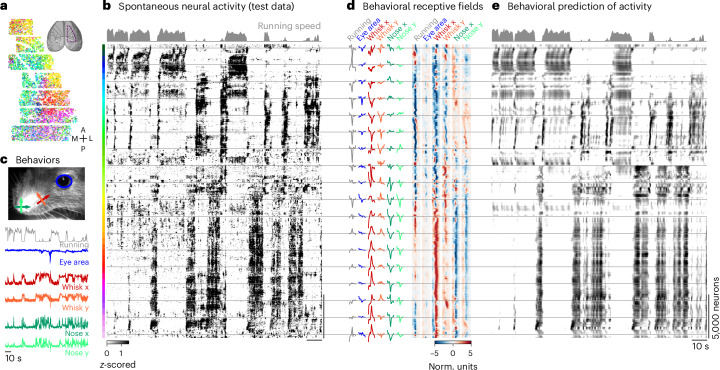
Sorting of spontaneous activity by Rastermap. **a**, In total, 34,086 neurons were recorded across mouse sensorimotor cortex using two-photon calcium imaging, colored by position in the Rastermap sorting. **b**, Neural activity sorted by Rastermap. **c**, Mouse orofacial behaviors during the recording. **d**, Left: example behavioral receptive fields for the superneurons at the Rastermap positions represented by the gray lines. Right: behavioral receptive fields for all superneurons in the Rastermap. **e**, Prediction of neural activity using the behaviors in **c**. The same Rastermap sorting of neurons as in **b** was maintained. Norm. units, normalized units.

Next, we applied Rastermap to data collected during spontaneous neural activity, where the animal is head fixed on top of an air-floating ball in complete darkness, without any explicit task^16,41^. In this preparation, we used a long ‘D’-shaped coverslip that covers many different cortical areas, including the anterior part of visual cortex, the sensorimotor cortex and the posterior part of motor cortex (Fig. 3a). In this case, we wanted to emphasize the global structure of population activity and did not observe sequential activity. Thus, we set the locality parameter to zero (Extended Data Fig. 8b). In general, we note that the locality parameter controls the balance between reproducing more of the global structure and more of the local structure in the data. Neurons across the brain had some degree of spatial clustering, as can be seen by their average position in the Rastermap sorting, indicated by the color of the dots. Over a period of 2 min, the populations of neurons visible in Rastermap engaged in a variety of activation patterns that lasted from hundreds of milliseconds to tens of seconds, with many of the patterns repeatable within this time window (Fig. 3b). Overall, the patterns could be divided into roughly two classes based on whether they were active during running or during sitting^42,43^, but, within those classes, different subsets of neurons were active at different times.

We previously showed that many of these spontaneous activity patterns can be predicted based on the orofacial behaviors of the mice, which we quantified either with a PCA-based decomposition of the face motion energy or by tracking keypoints on the mouse face^16,41^. To help us interpret the spontaneous activity clusters, we computed the eye area, whisker position and nose position estimates from the mouse face video using keypoint tracking (Fig. 3c)^41^. We then used these behavioral variables to estimate the spatiotemporal linear receptive fields for each superneuron from Rastermap and also to predict the superneuron activity across time (Fig. 3d,e). The receptive field is the spatiotemporal pattern of keypoint movements that would activate that particular superneuron the most. Across the Rastermap embedding dimension, the receptive fields change gradually and appear to be organized hierarchically, in which subsets of neurons with the same global response patterns have differential responses at more local timescales (Fig. 3d).

**Extended Data Fig. 10:**
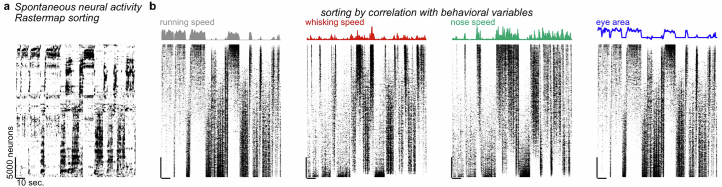
Rastermap sorting in comparison to behavioral-based sorting. **a**, Rastermap sorting of neural activity shown in Fig. 3b. **b**, Each neuron in the recording was correlated to the specified behavioral variable, and the neural activity was sorted by the correlation values.

The keypoint with the most influence on superneuron responses was the whisker horizontal location, which separates into negative deflections for the top clusters in the plot (that is, forward whisker deflections) and positive deflections for the bottom clusters (that is, backward whisker deflections). Within the set of clusters with negative whisker deflections, a subset was activated positively by running, and a subset was activated negatively. Analyzing the patterns of responses on the Rastermap plot itself, we observe different groups of neurons that are activated at the beginning and end of running, and those groups typically were inhibited by running in the model but excited by whisking. These neurons cannot easily be identified and visualized using the correlations to behavioral variables alone (Extended Data Fig. 10).

### Rastermap on other biological neural networks

We have so far illustrated Rastermap on large-scale calcium imaging data from mouse cortex. In this section, we show that Rastermap can be applied more broadly to recordings from other organisms, with fewer recorded neurons and even on bulk neural activity, such as from wide-field one-photon imaging. Finally, we show an application of Rastermap to artifical neural networks that are used to control agents that play Atari games.

**Fig. 4:**
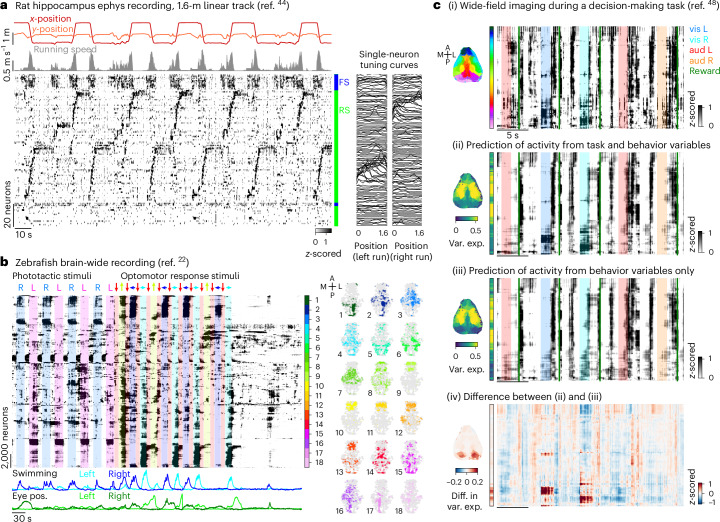
Visualizing neural activity across brain areas and species. **a**, Left: Rastermap sorting of neural activity collected from the CA1 region of rat hippocampus using two multi-shank silicon probes, during which the rat ran back and forth along a 1.6-m linear track^44^. Neuron identity was defined by spike waveform shape: FS, fast spiking (putative interneuron); RS, regular spiking (putative pyramidal cell). Right: tuning curves of each neuron to the position of the rat in the track. **b**, Left: Rastermap sorting of neural activity collected from the whole brain of a paralyzed zebrafish using light-sheet imaging at a rate of 2.1 Hz^22^. This period of time included two visual stimuli: phototactic stimuli (one side of the screen is dark) and optomotor response stimuli (moving gratings). Middle: color bar sectioning Rastermap into 18 groups for visualizing spatial patterning. Right: neuron positions in each group plotted in color; all neurons in the recording are in gray. Positions in each plot were collapsed across the *z* dimension. The color bar denotes the position along the embedding. **c**, (i) Rastermap sorting of cortical activity collected by wide-field imaging in mice performing a decision-making task^48^. The voxels in the brain image are colored by Rastermap position. Stimulus events in the task are indicated by colored shaded regions, and reward times are indicated by green lines. (ii) Linear prediction of activity from task and behavior variables shown with the same sorting as in (i). (iii) Same as (ii) with linear prediction from behavior variables only. (iv) Difference between prediction in (ii) and (iii). Eye pos., eye position; aud, auditory; vis, visual; var. exp., variance explained; diff. in var. exp., difference in variance explained.

When fewer neurons are recorded (<200), Rastermap can skip the *k*-means clustering step and directly order the neurons according to their asymmetric cross-correlogram peaks. This also allows us to skip the upsampling step, thereby simplifying the algorithm substantially. We applied this simplified version of Rastermap to electrical population recordings from rat hippocampus during running through a linear track (Fig. 4a)^44^. Rastermap found two main groups of neurons encoding forward and backward runs through the track. For each group, a subset of neurons encoded the stationary periods at the end of each run. Finally, another group of neurons had dynamics that were driven only by running and not selective to corridor position. This group turned out to be composed entirely of fast-spiking interneurons, which had relatively homogeneous activity.

Another use case for Rastermap is in multi-area or whole-brain recordings, such as from larval zebrafish using calcium imaging^45^. In this case, different groups of neurons may be identified that correspond to combinations of brain areas that perform a certain function together. We used recordings where different visual stimuli were presented (Fig. 4b)^22^: phototactic stimuli that elicited movement toward bright areas and drifting gratings that elicited optomotor responses toward the direction of the stimulus, primarily when the stimulus moved left and right. There were also periods in the recording with no visual stimulation, in which the fish rarely swam. Sorting with Rastermap, we found that many activity clusters correlated strongly with swimming in a condition-dependent fashion. Groups of neurons active during visually evoked swimming typically did not activate during spontaneous activity. However, the groups of neurons active during spontaneous activity were also often active during the visual stimulation conditions, although this activity was not aligned to the sensory stimulation events^15,46^. This resembles results that we previously found in rodent visual cortex^16^. These clusters were of two main kinds: (1) spread out throughout the fish brain and (2) concentrated in the anterior, forebrain areas. Another aspect of note was neuron clusters that were active for directional swimming regardless of condition (phototactic or drifting), whereas other clusters were only tuned to swim direction for specific conditions. These clusters generally aligned to sensory (frontal) and motor (posterior) areas in the fish brain, but substantial regions of overlap existed as well. Similarly, brain lateralization was apparent for most motor-related clusters, but some neuron groups from the other hemisphere were also sometimes included.

Rastermap can also be used on bulk signal recordings, such as from wide-field, one-photon calcium imaging in mice^47^. With this method, signals can be recorded from across the entire rodent cortex but not at single-cell resolution. Instead, each pixel may correspond to the averaged population activity at that location. We used wide-field recordings collected while the mouse performed a decision-making task and during which several behavioral variables were monitored (Fig. 4c)^48^. Because different cortical areas can engage for different behaviors, Rastermap can group together brain areas according to the similarity of their dynamics. As expected, the grouping had well-defined spatial relations (Fig. 4c, i). To start, the embedding was symmetric across hemispheres, with the left and right brain areas embedded at similar locations. Second, the most anterior pixels corresponded to the olfactory bulb and can be seen to have substantially different patterns of activity, which may be linked to sniffing bouts. The more posterior pixels also had different patterns of activity (corresponding to pink and red hues in the plot), and these may have been grouped together by visual responsiveness. To test this hypothesis, we predicted the pixel activities from task and behavior variables and found that these variables alone can explain activity across the entire Rastermap, including the anterior pixels in olfactory areas (Fig. 4c, ii). We compared this prediction to the prediction using behavior variables alone, which does not include information about stimuli (Fig. 4c, iii). The main difference between these two predictions was during visual stimulus trials, specifically in the visual cortex (Fig. 4c, iv).

### Trial-based analyses with Rastermap

**Fig. 5:**
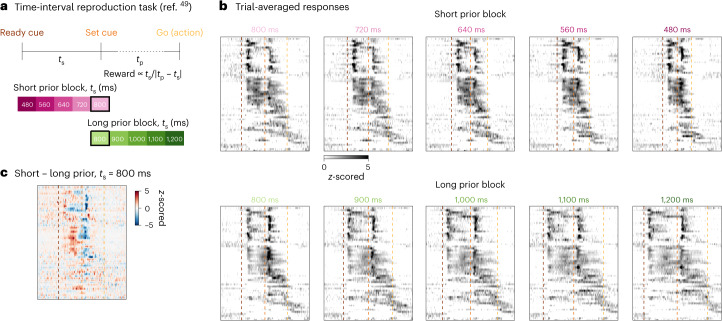
Visualizing monkey DMFC activity during a timing task. **a**, Top: two visual cues were flashed, indicating a ‘ready’ and a ‘set’ signal. The monkey needed to reproduce the time interval between these cues using its own ‘go’ action (saccade or joystick movement). Bottom: short and long trial blocks^49^. **b**, PSTHs for neurons sorted by Rastermap. The PSTHs of each neuron were *z*-scored together across conditions for visualization. **c**, The difference in neural responses between the top and bottom panels in **b** corresponding to the same time interval in blocks with different priors. Colored vertical dashed lines denote ready cue time, set cue time and go (action) time that maximizes reward. The color bar shows the difference in *z*-scored activity. *t*_s_, sample interval; *t*_p_, production interval.

So far, we have illustrated Rastermap on continuous-time data. However, many experiments have a trial-based structure that can be taken advantage of. In this section, we illustrate Rastermap on trial-based data from two electrophysiology datasets: one from monkeys performing a timing task and one from mice performing a discrimination task. In the timing task, monkeys had to estimate the duration of an interval spanned by two cues. There were short and long prior blocks, in which the intervals were drawn from a distribution with either short or long durations, respectively (Fig. 5a)^49^. We applied Rastermap on a dataset from this study of 54 simultaneously recorded neurons from the dorsomedial frontal cortex (DMFC), skipping the clustering step. Rastermap ordered neurons primarily based on their latency and response durations relative to the visual cues (Fig. 5b). As in ref. ^49^, we found neurons with differential activity depending on the block type (Fig. 5c), especially in the subset of neurons with sustained responses to the first cue.

**Fig. 6:**
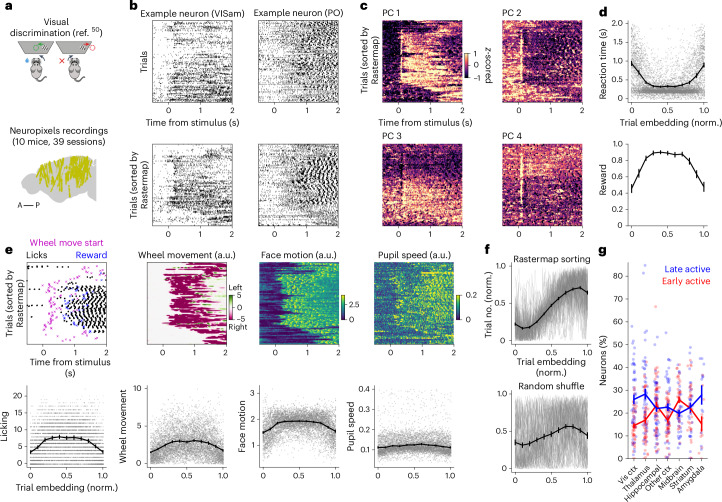
Sorting trials in a visual discrimination task. **a**, Top, mice were trained to discriminate between left and right contrast and to turn a wheel to report their choice (diagram from ref. ^61^). Bottom, locations of all neurons used, projected onto the sagittal plane (brain slice shape from ref. ^62^). **b**, Two example neuron trials sorted by time (top) or by Rastermap (bottom). **c**, PCs sorted by Rastermap for the same example recording as used in **b**, in z-scored units. **d**, Quantification of average reaction time and reward in the Rastermap sorting across sessions (error bars are s.e.m.; *n* = 78 sortings for left/right trials in 39 sessions). *x* axis represent trial index in Rastermap ordering, divided by total number of trials. **e**, Behavioral variables illustrated using the trial order obtained by Rastermap on the neural activity in an example session (top) and quantified across all sessions (bottom), in arbitrary units. Error bars are s.e.m. (*n* = 78). **f**, Trial number in session versus trial index in Rastermap ordering (top) and for a time-rolled shuffle of the ordering (bottom). The Rastermap order was flipped if the first 10 trials were on average later than the last 10 trials, and the same operation was applied to the shuffle. Error bars are s.e.m. (*n* = 78). **g**, Fraction of neurons with differential responses on trials early versus late in the session. Error bars represent s.e.m. (*n* = 50, 54, 58, 56, 40, 34 and 12 per brain region). norm., normalized (normalized by total number of trials); ctx, cortex.

Another option for trial-based data is to use Rastermap for sorting trials according to their similarity. We illustrate this on data from an experiment in which mice performed a two-alternative task, during which neural activity was recorded from up to 500–1,000 neurons at the same time from multiple brain regions^50^ (Fig. 6a). We sorted trials with the same motor action (that is, all right turns) using Rastermap and visualized single neurons or principal components (PCs) after sorting (Fig. 6b,c). The neural activity patterns aligned to the stimulus suggested that reaction time might be a substantial factor in the ordering, which we found to be the case, with longer reaction time trials placed both at the start and end of the Rastermap sorting (Fig. 6d). These types of trials also resulted in overall smaller rewards. Next, we wanted to investigate what distinguishes the two types of long-reaction-time, low-success-rate trials that Rastermap placed at the start or end of the Rastermap. We did not find a clear difference in behavioral variables, such as licking, wheel movement, face motion or pupil speed (Fig. 6e). However, we did find that the start and ending blocks of Rastermap trials also generally corresponded to the start and ending of the session (Fig. 6f). With this insight, we hypothesized that the neurons recorded had differential activity between the start and end of the session, such as from decreasing motivation and satiety. We found that, indeed, a large proportion of neurons in all brain areas were modulated in this way, with generally more neurons late-active in the session rather than early-active (Fig. 6g).

These initial results obtained with Rastermap provide a possible bridge between the brain-wide satiety signals reported in ref. ^51^ and the brain-wide sensory, decision and motor signals reported in ref. ^50^, both studies having been conducted with Neuropixels electrodes. Such exploratory analyses can provide a starting point for more in-depth exploration of the differences between early and late trials.

### Rastermap applied to artificial neural networks

**Fig. 7:**
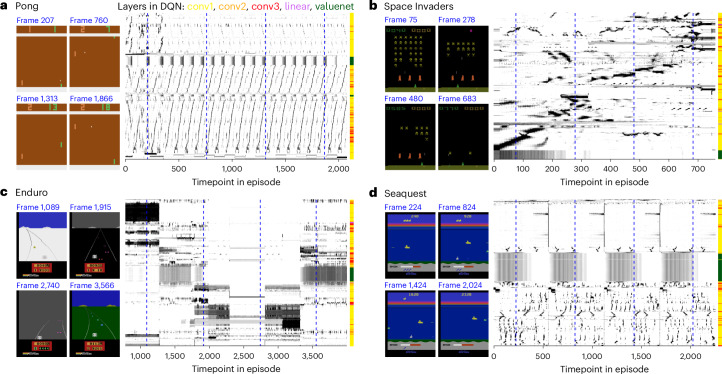
Applying Rastermap to artificial neural networks. We visualized activations from QR-DQNs that were trained to play various Atari games^52^. Superneurons were *z*-scored across the recording; white represents 0, and black represents 2.5. **a**, An agent trained on the Pong Atari game. Left: example frames from an episode of the agent playing Pong. Middle: activations of units from the agent’s neural network during the episode, sorted by Rastermap. Right: positions in the sorting colored by the layer in the network. **b**–**d**, Same as **a**, for agents trained to play Space Invaders (**b**), Enduro (**c**) and Seaquest (**d**). Blue vertical dashed lines denote the times of example frames illustrated in the left panels.

Finally, we also ran Rastermap on artificial neural networks that have been trained with reinforcement learning techniques to play Atari games (Fig. 7). We used pre-trained networks from Deep Q-Network (DQN) agents^52^ and clustered all neurons from across all layers of the DQN, illustrating four example games: Pong, Space Invaders, Enduro and Seaquest. In all cases, an episode consisted of a single playthrough of the respective game. In games with more repetitive action sequences, such as Pong, Rastermap found the repeated neural sequences that corresponded to each repetition and separated the forward portion of the sequence (ball moving right) from the backward portion (ball moving left). The details of each volley were encoded in the finer details of the neural activity. For games with less structured states, such as Space Invaders and Seaquest, Rastermap still found sequences of neurons that tend to activate together, but these sequences were more disorganized. In the case of the Enduro game, neural activation patterns were dominated by the graphical context of the game, which changed from day to night and between weather conditions, such as ‘fog’ and ‘ice’. Within each graphical state, mostly non-overlapping groups of neurons were active. A small set of neurons was active in more than one context, and these were generally found in the higher, more ‘abstract’ layers of the deep neural network. In all games, the neurons from the value network were placed all together in the Rastermap and appeared to have very homogeneous activity that directly corresponded to the value of a state. This indicates that perhaps the value network did not get sufficient gradient information to differentiate the activity of its neurons.

### Space-filling curves for higher intrinsic dimensionality

**Fig. 8:**
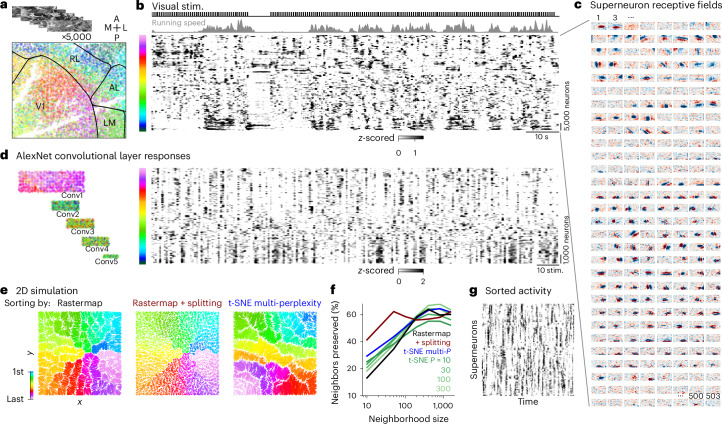
High-dimensional responses of real and artificial neurons to natural images and simulated two-dimensional activity. **a**, In total, 5,000 natural images were shown to a mouse during a two-photon calcium imaging recording from V1 and higher visual areas. **b**, Activity from 69,957 neurons was sorted by Rastermap with splitting and binned into superneurons, plotted with the mouse’s running speed and the visual stimulus times. **c**, Linear receptive fields for the superneurons in **b** in the same order, in arbitrary units. **d**, AlexNet convolutional layer responses to the same 5,000 natural images sorted by Rastermap with splitting. Left: units in the convolutional layers colored by the Rastermap sorting. Right: unit activations sorted and binned into superneurons shown across stimuli. **e**–**g**, We simulated neural activity with an intrinsic dimensionality of 2 by randomly choosing an *x* and *y* value for each neuron in the range of 0 to 1 and modeling its activity as a place field. **e**, Left: simulated neurons are plotted at their ground truth (*x*,*y*) positions and colored by their position in the Rastermap sorting run with *N*_clusters_ = 100. Middle: same as the left panel, using Rastermap with splitting, resulting in *N*_clusters_ = 800. Right: same as the left panel, using t-SNE with multiple perplexities (*P* = (10, 100)) to sort the neurons. **f**, The *k*-nearest neighbor score for benchmarking embedding algorithms from ref. ^26^: the percentage of *k*-nearest neighbors in the original space that are preserved as *k*-nearest neighbors in the embedding space—shown for Rastermap, Rastermap with splitting and t-SNE with various perplexities. **g**, Simulated activity sorted by Rastermap with splitting. RL, rostrolateral; AL, anterolateral; LM, lateromedial; V1, primary visual cortex; stim., stimulus; 2D, two-dimensional.

Rastermap is primarily a visualization algorithm, but visualizations can sometimes be deceptive, especially when the source data are high dimensional. In this section, we illustrate some use cases where Rastermap is ineffective at finding structure, and we try to provide an intuitive understanding of such cases. For example, we investigated Rastermap applied to neural responses in primary visual cortex to a large set of natural images (Fig. 8a). Natural images drive very high-dimensional response patterns across cortex, as we previously described^17^, and, thus, such data cannot be well described along any one-dimensional embedding dimension. Indeed, we observed that the Rastermap sorting had a high-dimensional, un-clustered aspect, except for some modulation induced by running (Fig. 8b). The running-modulated clusters corresponded primarily to neurons in higher-order visual areas that have less sensory tuning (Fig. 8a,b). By computing the linear receptive fields of superneurons from the Rastermap, we observed that nearby superneurons do, in fact, have similar receptive fields despite their apparently unorganized responses in the Rastermap (Fig. 8c). However, these receptive fields cannot be described by a single one-dimensional parameter, requiring several parameters to be well described: their retinotopic coordinates, their orientation, their spatial frequency, etc. To arrange filters with these properties across a one-dimensional continuum, Rastermap has to fill up this high-dimensional space with a so-called ‘space-filling curve’. Similar conclusions can be reached when applying Rastermap to the visual responses of an artificial deep neural network (Fig. 8d).

To better illustrate when ‘space-filling curve’ behavior occurs, we constructed a simulation where the underlying intrinsic dimension was 2, and neurons were described with place-field-like responses. Sorted with one-dimensional Rastermap or t-SNE, the neurons were arranged across a curve that meandered in a fractal way to fill up the two-dimensional space (Fig. 8e). We also constructed a version of Rastermap where the sorting was further broken up iteratively into sub-segments that were sorted with Rastermap again. After three consecutive splits, the simulated population was split into 800 rather than just 100 clusters, and this, in turn, resulted in a higher resolution of the underlying space-filling curve. This higher resolution resulted in better metrics of percent neighbors preserved at small neighborhood sizes, without affecting the number of preserved neighbors at the larger neighborhood sizes (Fig. 8f). Thus, although this iterative version of Rastermap can improve on some metrics, it is unlikely to provide fundamentally better visualizations, because the fractal nature of the space-filling curve makes the visualizations non-intuitive (Fig. 8g). Although a two-dimensional embedding algorithm could be employed, the results of such an algorithm cannot then be used to make raster maps of neural activity.

Our recommendation in these cases is to recognize that Rastermap is fundamentally a dimensionality reduction method: clustered activity can be found and illustrated in the Rastermap, but higher-dimensional structure may be discarded and missed when it exists. We recommend using other approaches to find and illustrate such high-dimensional structure, such as constrained matrix decomposition techniques (NNMF, seqNMF, ICA, TCA, dPCA, sparse coding, GPFA and DataHigh) or nonlinear dimensionality reduction with multiple dimensions (t-SNE, UMAP, LFADS, pi-VAE and CEBRA)^5,53–60^.

## Discussion

Here we described Rastermap, a visualization method that can be used to find new, interesting patterns in large-scale neural data. Rastermap makes a two-dimensional plot of neural activity versus time, allowing the user to observe complex spatiotemporal dynamics in relation to experimental events. At the core of the method lies a sorting algorithm that reorders the (possibly) tens of thousands of neurons so that nearby neurons in the sorting have similar activity. The sorting algorithm of Rastermap can also be seen as a one-dimensional embedding method and has several model features that allow it to accurately embed neural data: (1) modeling the long-tailed decay of pairwise correlations between neurons; (2) modeling sequential activity patterns that are often seen in neural data; and (3) using a specialized optimization algorithm that can avoid local minima. These features allow Rastermap to perform better as an embedding algorithm compared to other methods, such as t-SNE and UMAP, specifically in the case of one-dimensional embeddings for neural-like datasets.

Using Rastermap, we identified, for example, different groups of neurons in mouse sensorimotor areas corresponding to whisking at the onset of running and to whisking at the offset of running. We also found diverse patterns of activity associated with corridor positions and reward times in mouse visual areas during a virtual reality task. Rastermap applied to rat hippocampal activity revealed the structure of neural firing along a linear track in putative inhibitory and excitatory neurons. In zebrafish brain-wide activity, we observed lateralized and non-lateralized activity patterns associated with different motor and stimulus events. Rastermap sorting of wide-field neural imaging provided an unsupervised parcellation of the entire cortex, in part according to the predictability of different regions from different task variables. In monkey DMFC, we visualized neurons with specific tuning for the task block structure in a time-interval reproduction task. Using Rastermap to sort trials, we found two different types of long-reaction-time trials in a sensory decision-making task, and these putatively corresponded to different motivation states at the beginning and end of a session. We found that Rastermap could also be used to discover structure in artificial neural networks, such as those trained to play Atari games. Finally, using an extension of Rastermap, we explored datasets with higher intrinsic dimensionality, illustrating the limitations of low-dimensional embedding algorithms when applied to such datasets.

We hope that Rastermap will be applied to diverse dataset types, and we include a graphical user interface so that users can easily run the algorithm and explore their data. We consider Rastermap to be a good first step in examining neural population activity, such as when a new dataset is first obtained. Rastermap can help users find patterns in data, but, to fully demonstrate these patterns, appropriate quantitative analyses must be set up afterwards.

## Methods

All experimental procedures were conducted according to the Institutional Animal Care and Use Committee (IACUC) at Howard Hughes Medical Institute (HHMI) Janelia Research Campus.

### Data acquisition

#### Animals

All experimental procedures were conducted according to the IACUC; ethics approval was received from the IACUC board at HHMI Janelia Research Campus. We performed three recordings in three mice bred to express GCaMP6s in excitatory neurons: TetO-GCaMP6s × Emx1-IRES-Cre mice (available as RRID: IMSR_JAX:024742 and RRID: IMSR_JAX:005628). These mice were male and female and ranged from 2 months to 12 months of age. Mice were housed in reverse light cycle and were pair-housed with their siblings before and after surgery. Holding rooms were set to a temperature of 70 °F ± 2 °F and relative humidity of 50% ± 20%.

#### Surgical procedures

Surgeries were performed in adult mice (P35–P125) following procedures outlined in ref. ^32^. In brief, mice were anesthetized with isoflurane while a craniotomy was performed. Marcaine (no more than 8 mg kg^−1^) was injected subcutaneously beneath the incision area, and warmed fluids + 5% dextrose and buprenorphine 0.1 mg kg^−1^ (systemic analgesic) were administered subcutaneously along with dexamethasone 2 mg kg^−1^ via intramuscular route. For the visual cortical windows, measurements were taken to determine bregma–lambda distance and location of a 4-mm circular window over V1 cortex, as far lateral and caudal as possible without compromising the stability of the implant. A 4 + 5-mm double window was placed into the craniotomy so that the 4-mm window replaced the previously removed bone piece and the 5-mm window lay over the edge of the bone. The sensorimotor window was also a double window, and it was placed as medial and frontal as possible. The outer window was 7 mm × 4.5 mm, and the inner window was approximately 1 mm smaller in all dimensions. After surgery, ketoprofen 5 mg kg^−1^ was administered subcutaneously, and the animal was allowed to recover on heat. The mice were monitored for pain or distress, and ketoprofen 5 mg kg^−1^ was administered for 2 d after surgery.

#### Imaging acquisition

We used a custom-built two-photon mesoscope^35^ to record neural activity and ScanImage^63^ for data acquisition. We used a custom online *z*-correction module (now in ScanImage) to correct for *z* and *x*–*y* drift online during the recording. As described in ref. ^32^, for the visual area recordings, we used an upgrade of the mesoscope that allowed us to approximately double the number of recorded neurons using temporal multiplexing^36^.

The mice were free to run on an air-floating ball. Mice were acclimatized to running on the ball for several sessions before imaging, and one mouse was trained on a virtual reality task for 2 weeks before the recording. The field of view was selected such that large numbers of neurons could be observed, with clear calcium transients.

#### Visual stimuli

We showed natural images or virtual reality corridors to the mice on three perpendicular LED tablet screens surrounding the mouse (covering 270° of the visual field of view of the mouse). To present the stimuli, we used PsychToolbox-3 in MATLAB^64^. The flashed visual stimuli were presented for 313 ms, alternating with a gray screen inter-stimulus interval lasting 313 ms. Occasionally, the screen was left blank (gray screen) for a few seconds. The virtual reality corridors were each 4 m long, and the mouse moved forward in the virtual reality by running.

#### Videography

The camera setup was similar to the setup in ref. ^16^. A Thorlabs M850L3 (850 nm) infrared LED was pointed at the face of the mouse to enable infrared video acquisition in darkness. The videos were acquired at 50 Hz using FLIR cameras with a zoom lens and an infrared filter (850 nm, 50-nm cutoff). The wavelength of 850 nm was chosen to avoid the 970-nm wavelength of the two-photon laser while remaining outside the visual detection range of the mice^65,66^.

#### Processing of calcium imaging data

Calcium imaging data were processed using the Suite2p toolbox^67^, available at https://github.com/MouseLand/suite2p. Suite2p performs motion correction, region of interest (ROI) detection, cell classification, neuropil correction and spike deconvolution as described previously^16^. For non-negative deconvolution, we used a timescale of decay of 1.25 s^68,69^.

### Rastermap algorithm and implementation

The Rastermap algorithm is implemented in Python 3 using the NumPy, SciPy, numba and Scikit-learn packages, all of which are easy to install on Windows, Linux and Mac operating systems^31,70–72^. The graphical user interface is implemented using PyQt5 and PyQtGraph^73,74^. To perform analyses and create the figures in this paper, we used Jupyter notebooks and Matplotlib^75,76^.

The algorithm involves five main steps: dimensionality reduction, clustering, computing the asymmetric similarity matrix, sorting this matrix and upsampling the cluster centers. See Fig. 1a–d for a graphical representation of these steps. The input to the algorithm is a neural activity matrix of size neurons by timepoints. For electrophysiological datasets, we converted spike times into timepoints by binning in time.

#### Data normalization and dimensionality reduction

First, we normalize the neural activity to avoid fitting single-neuron statistics with the embedding algorithm. We z-score the activity of each neuron so that the mean activity of each neuron is zero and its standard deviation is 1. z-scoring can make low-firing neurons appear more active, but we have not seen issues with this in our applications, in which we use neurons with a minimum firing rate of 0.1–0.25 Hz. Next, we project out the mean across neurons at each timepoint; this is optional but is recommended (parameter mean_time in the algorithm). Note that, for some applications, this step may need to be skipped, for example if the grand average population activity is of interest. An optional step, which depends on the noise level of the data, is binning in time, parameter time_bin. These binned data are then used to compute the singular vectors.

Next, to make the data size more manageable and to speed up the clustering, we compute the singular value decomposition of this normalized activity matrix, where the left singular vectors will be of length number of neurons. We generally keep the top 100 to 400 left singular vectors; this can be specified by the user with the parameter n_PCs in the algorithm. We scale each of these singular vectors by its singular value, to preserve distances in the original space, and compute clusters from these scaled singular vectors. Thus, the matrix that we cluster is of size number of neurons by n_PCs, which is faster to cluster than a matrix of size number of neurons by timepoints.

#### Clustering

We clustered the neural activity PCs, defined as above, to create groups of co-active neurons. We clustered the neurons using scaled *k*-means clustering^67^. Compared to regular *k*-means, scaled *k*-means fits an additional variable *λ*_*i*_ for each neuron *i* such that

$${{{\bf{x}}}}_{i}={\lambda }_{i}{\mu }_{{\sigma }_{i}}+{{\rm{noise}}}$$

where **x**_*i*_ is the activity vector of neuron *i*; *σ*_*i*_ is the cluster assigned to neuron *i*; and *μ*_*j*_ is the activity of cluster *j*. Like regular *k*-means, this model is optimized by iteratively assigning each neuron to the cluster that best explains its activity and then re-estimating cluster means. The number of clusters *N* computed is called the n_clusters parameter in the algorithm.

#### Asymmetric similarity matrix

For each cluster out of *N* clusters, we compute the mean cluster activity by averaging all of the neurons in a cluster, and then we *z*-score each cluster activity trace. We then compute the cross-correlation between all cluster activity traces *c*_*i*_:

$$({c}_{i}* {c}_{j})[\tau ]=\frac{1}{T}\mathop{\sum }\limits_{t=0}^{T}{c}_{i}(t-\tau ){c}_{j}(t).$$

This is computed for a specified number of positive *τ* time lags ${\tau }_{\max }$, called the time_lag_window parameter in the algorithm. Then, we use the maximum value of the cross-correlation for each cluster pair over these positive *τ* values for our asymmetric similarity matrix *S*:

$${S}_{i,\;j}=\mathop{\max }\limits_{\tau \in [0,{\tau }_{\max }]}({c}_{i}* {c}_{j})[\tau ]$$

Matrix *S* is of size *N* by *N*, where *N* is the number of clusters.

#### Sorting the similarity matrix

We optimize the sorting of the asymmetric similarity matrix of the cluster nodes to maximize a matching score. This score is defined as the dot product between the sorted version of the asymmetric similarity matrix *S*^sorted^ and a pre-specified matching matrix *M*, which is the same size as *S*:

$${{\rm{Score}}}=\mathop{\sum}\limits_{i}\mathop{\sum}\limits_{j}{M}_{i,\;j}{S}_{i,\;j}^{{{\rm{sorted}}}}$$

This matching matrix has two parts: a global similarity and a local ‘traveling salesman’ similarity. The global similarity matrix is defined as

$${M}_{i,\;j}^{{\,{\rm{global}}}}=-\log (| {x}_{i}-{x}_{j}| +0.001)$$

where *x*_*i*_ = *i*/*N*, and the diagonal ${M}_{i,i}^{{\,{\rm{global}}}}$ is set to zero. This type of matrix can be shown to have an approximately power law decay of eigenvalues with exponent 1 (ref. ^17^). We define *M*^global^ to have a heavy-tailed power law decay to approximate the high-dimensional structure of neural activity observed in various contexts^15–18^.

The local similarity matrix is close to 1 on the first off-diagonal and very small elsewhere:

$${M}_{i,\;j}^{{{\rm{local}}}}=\exp (-{({x}_{i}-{x}_{j})}^{2}/(2{\sigma }^{2}))$$

where *σ* = 1/(2*N*), and the diagonal ${M}_{i,i}^{{{\rm{local}}}}$ is set to zero. We define *M*^local^ to have large values near the diagonal to force clusters with high correlations to be put next to each other, preserving local correlations.

We then set the lower diagonal of each matrix to zero to force all correlations to be put above the diagonal, which enforces forward sequences of activity. Each of these matrices is then normalized by its mean across all entries. Then, the final matching matrix is a weighted sum of the two matrices:

$$M=(1-w){M}^{{{\rm{global}}}}+w{M}^{{{\rm{local}}}}$$

where the weighting *w* is called the locality parameter in the algorithm. *w* can vary from 0 to 1.

We initialize the sorting by the first singular vector weights for each cluster node. Then, we compute the change in score for each cluster moved to each position in the matrix (n × (n − 1) tested moves). We first test all movements of groups of a single node, and then we move the node that increases the score the most. If none of the moves will increase the score, then we test all moves of two consecutive nodes, and, similarly, if none of those moves, we test all groups and moves of three nodes and so on. We repeat each step of searching for moves of groups of nodes that increase the score for 400 iterations or until no move of any group of nodes increases the score. During this optimization, we skip every other node if the number of clusters to sort is 100 (the number of nodes skipped is the floor of the number of clusters divided by 30). If nodes were skipped, then the optimization is run again with all nodes for up to 400 iterations, although it usually takes fewer than 10 iterations to converge during this second run, and, thus, skipping nodes reduces runtime. This optimization can be made highly parallel: we can test all moves of groups of nodes of a certain length simultaneously and then choose the best move. Therefore, we accelerated this step and other steps in the optimization using the numba library^31^ in Python, which can be easily installed on any standard desktop or laptop computer.

This optimization takes less than 10 s for 100 clusters, approximately 20–30 s for 150 clusters and 1–2 min for 200 clusters. Because it exponentially gets slower for larger numbers of clusters, we find more positions for neurons by upsampling rather than using more clusters.

#### Upsampling and superneuron computation

We upsampled in-between cluster centers in PCA space using weighted, locally linear regression, to go from *N*_clusters_ to 10 × *N*_clusters_ nodes. The regression approximated linearly the function from discrete cluster index to PCA features, in small local neighborhoods around each cluster index. The sizes of these neighborhoods were controlled by weighting the regressors according to their index separation in the Rastermap sorting. The weightings were Gaussian as a function of Euclidean distance with standard deviation $\sigma =1/(\sqrt{2})$. Cluster centers beyond the 50 nearest neighbors of an upsampled point were not used. We performed this linear approximation at each upsampled position, at a resolution of 10× the original resolution of the data. The upsampled features were then correlated with each neuron activity, and neurons were assigned to the position of their best-matching upsampled node.

#### Clustering and splitting steps

To increase the number of clusters, we also explored a strategy inspired by space-filling curves^77^. Starting with 100 sorted clusters, we divide the sorting into quartiles of 25 clusters each and recluster the neurons in each quartile into 50 clusters. We then sort each of these groups of 50 clusters with the iterative optimization, including the asymmetric similarity matrix surrounding each group in the score to avoid discontinuities in the final sorted matrix across groups of clusters. The splitting and reclustering can be performed as many times as preferred; the parameter in the algorithm is n_splits. For the analyses in Fig. 8, we split three times, resulting in 800 total clusters.

### Simulations

To compare the performance of different embedding algorithms, we created simulations of large-scale data with noise (Figs. 1 and 8).

#### Simulation with different modules

We created a simulation with five different types of one-dimensional modules: two sequence modules, one module with tuning curves, one module with sustained stimulus responses and one module with power law eigenvalues (Fig. 1e). The first four modules had 1,000 neurons each, and the last module had 2,000 neurons.

Each neuron in a sequence module was assigned to a random position along the sequence at which point it activated. The sequences in the sequence modules repeated many times throughout the simulation, with each repetition having a random length between 350 and 700 timepoints and the time between repetitions having a random length between 100 and 200 timepoints. A variable velocity for each sequence repetition was generated by adding to a constant velocity random Gaussian noise filtered by a Gaussian with standard deviation 30 timepoints. These sequences could break at a random place for a short period with a probability of 50% per repetition, simulating the breaks in sequences that we observed when mice stopped moving through virtual corridors.

The tuning curve module consisted of neurons with one-dimensional Gaussian tuning curves at 1,500 possible positions along this axis. We presented, in a random order, 15 stimuli equally spaced along this one-dimensional axis. In total, there were 500 stimulus presentations spaced 100 timepoints apart. The stimulus responses from the neurons decayed exponentially from the onset with a timescale of 25 timepoints.

The sustained module consisted of neurons with varying latencies and response durations to a single stimulus generated using a difference of exponentials filter with 100 timescales ranging from (25, 5) to (304, 61). Each neuron in the modules was assigned randomly to one of these 100 timescales. The inter-stimulus interval was drawn from an exponential with a decay timescale of 750 timepoints, with a minimum value of 2,000 timepoints used.

The power law module consisted of a neural population with eigenvalues *e*_*k*_ = 1/*k*^1.5^. The right singular vectors of the neural population *V*, with length time, consisted of a sparse Boolean matrix filtered by an exponential filter with a timescale of 25 timepoints. The left singular vectors *U* were composed of cosine functions with increasing frequency as a function of component number *k*: $\cos (\pi kx)$, where *x* is a random number for each neuron between 0 and 1. Each neuron in the entire simulation is assigned a random *x* value. *U*, *S* and *V* were then multiplied and clipped at zero to create a positive neural activity matrix. We then added this activity to every neuron in the simulation with a weight of 0.75; this reproduced the property in the data that most neurons are driven during spontaneous activity periods, and they continue to be driven by spontaneous activity patterns during stimulus presentations. In total, 2,000 neurons out of 6,000 were driven solely by the power law module.

The activity matrices created in each module represent the firing rate of each neuron at each timepoint. We scaled each firing rate trace by a random number drawn from an exponential to create neurons with different firing rates. We then used the Poisson distribution to generate spikes from these firing rates. We also added independent Poisson noise to all the neurons with mean 0.03. We sorted the neural activity with Rastermap using n_clusters = 100, n_PCs = 200, locality = 0.8 and time_lag_window = 10 in Fig. 1. We changed the random seed, which controlled the initialization for the scaled *k*-means clustering, using 20 different values in Extended Data Fig. 6. We varied the number of clusters, the locality parameter and the time_lag_window parameter in Extended Data Fig. 7. When using the Leiden algorithm for clustering, we set the number of neighbors to 100 and the resolution to 3.0, which produced approximately 100 clusters (Extended Data Fig. 7a)^33,78^.

#### Simulation with power law module only

We created a power law module as above, consisting of 6,000 neurons, with eigenvalues *e*_*k*_ = 1/*k*. The activity matrices created in each module represent the firing rate of each neuron at each timepoint. We scaled each firing rate trace by a random number drawn from an exponential to create neurons with different firing rates. We then used the Poisson distribution to generate spikes from these firing rates. We sorted the neural activity with Rastermap using n_clusters = 100, n_PCs = 200, locality = 0 and time_lag_window = 0.

#### Simulation with intrinsic dimensionality of 2

We simulated neural activity with an intrinsic dimensionality of 2 by randomly choosing an *x* and *y* value for each neuron in the range of 0 to 1 (Fig. 8). We simulated 30,000 neurons in total using basis functions, which depended on *x* and *y*: ${U}_{{k}_{x},{k}_{y}}(x,y)=\cos (\pi {k}_{x}x)* \cos (\pi {k}_{y}y)$, where *k*_*x*_ and *k*_*y*_ compose a two-dimensional grid from 1 to 30, for a total of 900 basis functions, which we used as the left singular vectors for construction of the simulation. The singular values were defined as ${s}_{{k}_{x},{k}_{y}}={({k}_{x}^{2}+{k}_{y}^{2})}^{-0.5}$. The right singular vectors *V* were generated as random Gaussian noise at each of the 20,000 timepoints. We multiplied *U*, *S* and *V* and then added random Gaussian noise scaled by 5 × 10^−3^ at each timepoint. We sorted the neural activity with Rastermap using n_clusters = 100, n_PCs = 400, locality = 0, time_lag_window = 0 and n_splits = 0 or n_splits = 3 (resulting in 800 clusters due to splitting). We binned the sorted neural activity into superneurons of size 60 neurons each (Fig. 8g); the neurons are colored by the sorting in Fig. 8e.

### Benchmarking embedding algorithms

Each module in the one-dimensional simulation has a ground truth sorting defined by its one-dimensional axis. We compared the embedding order found by a given algorithm to this ground truth order using two metrics: the percent of correctly ordered triplets and the percent contamination of neuron groups from the same module. To compute the triplet score, we drew many groups of three random neurons from the same module, and, if these three neurons were in the same order in the embedding as in the ground truth, then it was considered a correct triplet. For the percent contamination, we drew many groups of two random neurons from the same module and quantified the percentage of neurons between these two neurons, which were from a different module, and averaged the percentage over all groups. The results are shown in Fig. 1i,j. For the simulation with only one module, the power law module, we did not need to use these more complicated metrics and, instead, simply correlated the algorithm sorting with the ground truth sorting and took the absolute value.

Another way to benchmark embedding algorithms is to quantify how well the local neighborhood of a data point in the original space is preserved in the embedding space^26^. This is done by computing the percentage of *k*-nearest neighbors in the original space are preserved as *k*-nearest neighbors in the embedding space. In our case, for the original space, we can use the ground truth position of the neuron in the simulation and compute distances between these positions rather than using noisy estimates of distances from the data as is required when the ground truth is unknown. We computed the percentage of neighbors preserved for *k* from 1 to 500 on a random subset of 2,000 neurons from the full simulation (to speed up the neighbor computation); the results are shown in Extended Data Fig. 5.

We also defined local and global preservation scores to benchmark the asymmetric similarity matrices from the data (Extended Data Fig. 8). The local score is defined as the fraction of first upper diagonal entries in the sorted asymmetric similarity matrix, which were the largest possible values in the matrix (similar to the neighbor preservation score). The global score is defined using the cost function from the matching matrix: the dot product between the upper triangular of the sorted asymmetric similarity matrix and the upper triangular of the matching matrix, normalized by the mean of the upper triangular of the matching matrix.

#### Running other embedding algorithms

We compared Rastermap to the most commonly used embedding algorithms: t-SNE^19^, UMAP^20^, ISOMAP^79^, Laplacian Eigenmaps^80^, hierarchical clustering^81,82^ and PCA (Fig. 1h–j and Extended Data Fig. 5a). We ran the openTSNE implementation of t-SNE due to its efficiency and flexibility^83^. We ran t-SNE and UMAP with the suggested initialization from ref. ^26^: the first PC scaled by a small number (we chose 0.0001). We used the cosine similarity metric for t-SNE, UMAP and ISOMAP, as this improved performance. We ran the ‘linkage’ and ‘fcluster’ methods in SciPy to perform hierarchical clustering, with the correlation similarity metric, the ‘single’ method and *t* = 0.01 as this improved performance^71^. Otherwise, the algorithms were run with their default parameters.

The performance of t-SNE and UMAP can depend on their parameters that define their local neighborhoods, perplexity and n_neighbors. Therefore, we also ran t-SNE and UMAP with several different values of these parameters to determine whether they influenced the embedding quality (Extended Data Fig. 5b,c). For Extended Data Fig. 6, we ran t-SNE with 20 different random seeds, keeping the initialization fixed on each run to the first PC scaled by 0.0001.

### Data analysis

#### Neural activity from a virtual reality task

We analyzed neural activity collected from mouse visual cortical areas using two-photon calcium imaging at a rate of 3.2 Hz while the mouse was free to run on an air-floating ball in a virtual reality task (Fig. 2a). The task contained two virtual corridors, ‘leaves’ and ‘circles’, and the ‘leaves’ corridor was rewarded at a random position in the corridor after a sound cue (the sound cue was also played in the ‘circles’ corridor but not rewarded) (Fig. 2b). We sorted the neural activity with Rastermap using n_clusters = 100, n_PCs = 200, locality = 0.75 and time_lag_window = 10, and we binned the sorted neural activity into superneurons of size 100 neurons each (Fig. 2d); the neurons are colored by the sorting in Fig. 2a, and the asymmetric similarity matrix for the clusters is shown in Fig. 2c. The superneuron tuning curves were computed for 100 positions along each corridor and in the gray space between corridors.

#### Spontaneous activity in sensorimotor areas

We analyzed neural activity collected from a large part of mouse dorsal cortex using two-photon calcium imaging at a rate of 3.2 Hz, centered on sensorimotor areas, while the mouse was free to run on an air-floating ball in total darkness (Fig. 3a). We sorted the neural activity with Rastermap using n_clusters = 100, n_PCs = 128, locality = 0.0 and time_lag_window = 5, and we binned the sorted neural activity into superneurons of size 50 neurons each (Fig. 3b); the neurons are colored by the sorting in Fig. 3a.

We used the keypoint tracking network from ref. ^41^ to track keypoints on the mouse face from the video taken during the recording (Fig. 3c). From these keypoints, we computed five interpretable variables: the eye area, the whisker pad position and the nose position. The eye area was computed by taking the difference of the top and bottom eye keypoints and the difference of the left and right eye keypoints and then multiplying these two values together. The whisker pad position was computed by averaging the positions of the three tracked whisker keypoints. Then, the PCs of the *x* and *y* positions were computed and used to rotate the coordinates such that the new *x* position corresponded to movements along the major axis of whisker movements. The nose position was computed by averaging the position of the four tracked nose keypoints.

Next, we used the neural network from ref. ^41^ to predict 128 neural activity PCs from these five variables and the running speed. The behavioral prediction from this nonlinear neural network was visualized in Fig. 3e. To estimate the superneuron receptive fields, we used a simplified linear version of this neural network. The network consisted of the same first two layers, an input linear layer and a one-dimensional convolutional layer, and then these layers were followed by a single output linear layer, which predicted the 128 PCs (Fig. 3d). The receptive field for a superneuron was estimated by optimizing a small behavioral snippet of length 8 s to maximally activate the superneuron at the timepoint at the midpoint of the snippet.

#### Rat hippocampus data

We analyzed a freely available neural activity recording collected from the CA1 region of rat hippocampus using two multi-shank silicon probes, during which the rat ran back and forth along a 1.6-m linear track (Fig. 4a)^44,84^. We binned the spiking in time bins of 200 ms, and we used the full time period in which the rat was in the maze and used all 137 recorded neurons. To estimate the location of the rat and the start and stop of the rat in each corridor, we used code available from ref. ^59^.

We sorted the neural activity with Rastermap using n_clusters = None, n_PCs = 64, locality = 0.1 and time_lag_window = 0. When n_clusters is set to None or to 0, then the algorithm sorts the original datapoints—the single neuron traces—rather than first clustering the data and then sorting. Tuning curves for leftward runs and rightward runs along the corridor were computed for 30 positions along the track.

#### Zebrafish whole-brain data

We analyzed a freely available neural activity recording collected from the whole brain of a paralyzed zebrafish using light-sheet imaging at a rate of 2.1 Hz (Fig. 4b)^22,85^. During the imaging session, the zebrafish was presented many different visual stimuli, such as phototactic stimuli (one side of the screen is dark) and optomotor response stimuli (moving gratings). The fictive swimming was recorded with electrodes, and the eye positions were tracked. We removed neurons that had low signal-to-noise ratio using a threshold of 0.008 on the fluorescence standard deviation. To remove long timescales from the calcium sensor in the data, we baselined the fluorescence traces and ran non-negative spike deconvolution with a timescale of 2 s^68,69^.

We sorted the neural activity with Rastermap using n_clusters = 100, n_PCs = 200, locality = 0.1 and time_lag_window = 5, and we binned the sorted neural activity into superneurons of size 50 neurons. We then divided the plot into 18 bins to color neurons across the fish brain by position (Fig. 4b, right).

#### Wide-field imaging data

We analyzed a freely available wide-field cortical imaging recording collected from mice performing a decision-making task (Fig. 4c)^48,86^. We discared voxels on the edges of the recording area as these voxels were noisy in time, but this step is optional and data dependent. In total, 186,590 voxels remained for analysis, by 93,177 timepoints—the data were collected at a rate of 30 Hz. Because wide-field imaging recordings are very large (hundreds of thousands of voxels by hundreds of thousands of timepoints), they are often summarized by their singular value decomposition. Rastermap has the option to run on these singular vectors alone rather than the full dataset. We sorted the voxel singular vectors with Rastermap using n_clusters = 100, n_PCs = 200, locality = 0.5 and time_lag_window = 10, and we binned the sorted voxels into supervoxels of size 200 voxels.

Next, we predicted the supervoxel activity from behavior variables or both behavior and task variables. These were pre-computed in ref. ^86^ in ‘regData.mat’. The behaviors used were handle-grabbing movements, licking, whisking, nose movements, filtered pupil area, face movements, body movements and PCs of the raw video of the mouse and the video motion energy. The task variables used were reward times, choice, previous choice, water delivery, piezo, visual stimuli and auditory stimuli. We *z*-scored each of these variables across time. We predicted the voxel activity from these variables using linear regression with a regularization constant of 1 × 10^4^. The prediction from behavior-only is shown in Fig. 4c (ii), and the prediction from behavior and task variables is shown in Fig. 4c (iii). The difference between the two predictions is shown in Fig. 4c (iv).

#### Time-interval reproduction task data

We analyzed neural activity recorded in DMFC from a monkey performing a time-interval reproduction task (Fig. 5a)^49,87^. This dataset is provided in the Neural Latents Benchmark^88^. We used all 54 neurons and binned the neural activity in 20-ms bins. The neural activity across all timepoints was sorted by Rastermap with no clustering, n_PCs = 48, time_lag_window = 20 and locality = 0.5. The peristimulus time histogram (PSTH) for each neuron and for each trial type was computed aligned to the set cue time. The PSTHs of each neuron were z-scored together across all trial types for visualization (Fig. 5b). The go (action) time was plotted as the average action time across all trials of a given time. The 800-ms interval trials were subtracted from each other to compare the average responses (Fig. 5c).

#### Visual discrimination task data

We analyzed neural activity collected from 10 different mice across 39 recording sessions, acquired while they performed a visual discrimination task in which decisions were reported by turning a wheel (Fig. 6a)^50,89^. We used all neurons with an average firing rate of at least 0.1 Hz across all trials, resulting in 25,906 neurons in total across all sessions. Neural activity was binned in bins of size 10 ms, and trials were defined as from 500 ms before the stimulus presentation to 2 s after the presentation (using the formatting from Neuromatch^90^). From each session, we computed the top 10 PCs of the neural activity. We split the trials into right-turn and left-turn trials, excluding trials in which the mouse did not turn the wheel. We sorted the PCs of each of these trial types, concatentating the PCs in time, resulting in a matrix of size number of trials by (10 × number of timepoints). The trial axes of these matrix were sorted by Rastermap with no clustering, n_PCs = 64 and locality = 0.1, and we set time_lag_window = 0 and mean_time = False because we are not sorting neurons over time here. This resulted in 78 sortings (two for each session for right-turn and left-turn trials). An example neuron and PCs with the right-turn trials sorted are shown in Fig. 6b,c, along with the behaviors of the mouse in these trials shown in Fig. 6e, top.

Reaction time (Fig. 6d) was defined as the time when the mouse first moved the wheel. The averages for all behavioral variables are shown in black, computed using 10 equally spaced bins (Fig. 6d,e). For Fig. 6f, top, we plotted the Rastermap sorting versus trial number; Rastermap sortings were flipped if the average trial number in the session for the first 10 sorted trials was greater than for the last 10 sorted trials. Shuffling was performed by circularly permuting each sorting by a random number and then flipping as in the top panel.

We computed the rank-sum difference between single-neuron firing rates on the first 20 and last 20 trials of the recording, for right-turn and left-turn trials separately. We used the Wilcoxon two-sided rank-sum test to define ‘late-active’ and ‘early-active’ neurons, using a significance threshold of *P* < 0.05. We computed the percentage of ‘late-active’ and ‘early-active’ neurons in each brain region per session and trial type, for all sessions in which the brain area was present (Fig. 6g).

#### Visual stimulus responses

We analyzed neural activity collected from a subset of mouse visual cortical areas using two-photon calcium imaging at a rate of 3.2 Hz while the mouse was free to run on an air-floating ball and grayscale natural images were presented (Fig. 8a). A natural image was shown on every other neural frame. There were 5,000 different images in total, presented three times in a random order. To compute linear receptive fields, we downsampled the natural images to size 24 × 96 and then computed the top 200 PCs.

We sorted the neural activity with Rastermap using n_clusters = 100, n_PCs = 400, n_splits = 3, locality = 0.0 and time_lag_window = 0 (resulting in 800 clusters due to splitting), and then we binned the sorted neural activity into superneurons of size 139 neurons to create 500 superneurons in total (Fig. 8b). We then averaged the responses of each superneuron over the three repeats of the 5,000 images. Using the averaged responses, we computed the linear receptive fields of each superneuron with linear regression from the image PCs with a regularization constant of 1 × 10^4^ (Fig. 8c).

### Neural network experiments

#### DQN playing Atari games

We analyzed the activations of neural networks trained to play Atari games, from the Stable-Baselines3 RL Zoo (Fig. 7)^91,92^. These networks were Quantile Regression DQNs (QR-DQNs), which consisted of three convolutional layers and a linear layer to process the images from the game (four frames stacked in time) and a feedforward network to compute the state values^52,93^. We used four different ‘NoFrameskip-v4’ agents each trained on a different environment: Pong, Space Invaders, Enduro and Seaquest. We ran the environments 10 times, each time with a different random seed, and then we concatenated the activations across the 10 episodes. Each episode lasted for up to 4,000 timepoints or however long until the agent won or lost in each run (only for the Enduro environment did this exceed 4,000 timepoints because the Enduro game can never be won, and the agent never lost).

We sorted the neural network activations across all 10 episodes with Rastermap using n_clusters = 100, n_PCs = 200, locality = 0.75 and time_lag_window = 10, and we binned the sorted activations into superneurons of size 50 units. We showed the activations for one episode along with four example frames in Fig. 7.

#### AlexNet in response to natural images

We trained the AlexNet neural network to perform image recognition on ImageNet images in grayscale (rather than the usual RGB)^94,95^. We then presented the network the same natural images that we showed to the mice and saved the activations in all of the layers. For further analysis, we used 2,560 random activations from the first four convolutional layers and all 1,280 activations from the fifth convolutional layer.

We sorted the AlexNet activations with Rastermap using n_clusters = 100, n_PCs = 400, n_splits = 3, locality = 0.0 and time_lag_window = 0 (resulting in 800 clusters due to splitting), and then we binned the sorted activations into superneurons of size 24 units (Fig. 8d, right). We colored each of the activations by their position in the Rastermap (Fig. 8d, left).

### Statistics and reproducibility

No statistical method was used to predetermine sample size. We found that the performance of the various algorithms was consistent across the 10 randomly generated simulations, suggesting that 10 random simulations were sufficient (error bars represent s.e.m. in Fig. 1) (many methods papers use only one randomly generated simulation^58–60^). We performed rank-sum tests to determine selective neurons in Fig. 6, which do not require the data to be normal. No data were excluded from the analyses. There were no experimental groups so no randomization was necessary. Data collection and analysis were not performed blinded to the conditions of the experiments.

### Reporting summary

Further information on research design is available in the Nature Portfolio Reporting Summary linked to this article.

## Online content

Any methods, additional references, Nature Portfolio reporting summaries, source data, extended data, supplementary information, acknowledgements, peer review information; details of author contributions and competing interests; and statements of data and code availability are available at 10.1038/s41593-024-01783-4.

## Supplementary information

Reporting Summary

## Data Availability

All data used in this study are publicly available. The large-scale calcium imaging data are available at 10.17605/OSF.IO/XN4CM. Previously shared datasets were also used in this study and are available at 10.6080/K0862DC5, 10.25378/janelia.7272617.v4, 10.14224/1.38599, 10.48324/dandi.000130/0.220113.0407 and 10.6084/m9.figshare.9598406.v2 (refs. ^84–87,89^).

## Code availability

Rastermap was used to perform all analyses reported in this paper. The code and graphical user interface are available at https://www.github.com/mouseland/rastermap (ref. ^96^). Scripts for running all the analyses reported in this paper are available at https://github.com/MouseLand/rastermap/tree/main/paper.
